# The effect of surgical approach on early complications of total hip arthroplasty

**DOI:** 10.1186/s42836-019-0008-2

**Published:** 2019-09-03

**Authors:** Kenny Tay, Andrew Tang, Camdon Fary, Sam Patten, Robert Steele, Richard de Steiger

**Affiliations:** 1 Arthroplasty Fellow, Epworth Musculoskeletal Clinical Institute, 89 Bridge Road, Richmond, Melbourne, VIC 3121 Australia; 2 Fellowship of the Royal Australasian College of Surgeons, Epworth Musculoskeletal Clinical Institute, 89 Bridge Road, Richmond, Melbourne, VIC 3121 Australia; 3 Present Address: Department of Orthopaedic Surgery, Singapore General Hospital, Outram Road, Singapore, 169608 Singapore

**Keywords:** Total hip, Approach, Complications

## Abstract

**Background:**

Total hip arthroplasty (THA) is traditionally associated with a low complication rate, with complications such as infection, fracture and dislocation requiring readmission or reoperation. We seek to identify the complication rate among the anterior, direct lateral and posterior surgical approaches.

**Methods:**

We reviewed all THAs performed at the Epworth Healthcare from 1 July 2014 to 30 June 2016. There were 2437 THAs performed by a variety of approaches. No hips were excluded from this study.

We surveyed the hospital database and the Australian Orthopaedic Association National Joint Replacement Registry (AOANJRR) to identify those patients who had been readmitted and/or reoperated on. Details collected included age, gender, laterality of the surgery (left/right/bilateral), surgical approach utilised, complications which occurred.

**Results:**

There were 29 peri-prosthetic fractures detected (13 anterior, 9 lateral, 7 posterior) and 10 underwent revision of implant, 19 were fixed. The increased rate of revision in the anterior group was statistically significant.

There were 14 dislocations (5 anterior, 1 lateral, 8 posterior) of which 8 prostheses were revised.

Three cases operated via the anterior approach and 1 by the lateral had early subsidence without fracture, necessitating revision of the femoral prostheses.

Operative site infection occurred in 12 cases (2 anterior, 4 lateral, 6 posterior) with 6 requiring revision of implants.

**Conclusion:**

The complication rates between the 3 main approaches are similar, but individual surgeons should be vigilant for complications unique to their surgical approaches, such as femoral fractures in the anterior approach and dislocations in the posterior approach.

## Background

Total Hip Arthroplasty (THA) is generally associated with a low complication rate, ranging from 1 to 3%. Infection, peri-prosthetic fracture (PPF) and dislocation are early complications that may require re-admission to hospital for management. The surgical approach may also influence early outcomes. There are 3 commonly used approaches for the hip joint, the posterior, direct lateral and anterior. The latter approach has been used increasingly over the past few years with reported benefits including shortened hospital stay [1], reduced post-operative pain and a potential for reduced blood loss [2, 3]. However, recent literature has brought attention to the complication rate of the anterior approach, in particular iatrogenic femoral fractures, dislocations and wound complications [4, 5]. These have been known to be related to the learning curve for the procedure [6–8].

The posterior approach to the hip is the most common surgical approach used internationally for THAs [9]. This approach necessitates judicious posterior soft tissue repair in order to decrease the risk of post-operative dislocations. The lateral direct lateral approach, as described by Hardinge, has been used for many years and has a low reported dislocation [10]. Abductor insufficiency occurs in 4–20% of patients, which can result in a Tredelenburg gait [11]. There is relatively sparse literature comparing the complications of the 3 main approaches, as more attention has been paid to outcome comparisons between approaches.

The primary aim of this study was to determine if different surgical approaches to THA are associated with an increased complication rate requiring either re-admission or re-operation. The secondary aim was to see if complications were associated with an increased rate of revision.

## Methods

Epworth HealthCare is a private healthcare group in Victoria, Australia comprising a combined total of 1200 beds. Approximately 1200 THAs are performed annually. This study was approved by the Research Development & Governance committee of Epworth HealthCare. We reviewed the records of all primary THAs performed at Epworth HealthCare from 1 July 2014 to 30 June 2016. All surgeries were performed by experienced surgeons in private practice utilising three approaches, i.e., the posterior, direct lateral and anterior hip approaches.

Demographic details collected included age, gender, laterality of the surgery (left/right/bilateral), surgical approach utilised, complications, number of and time to reoperations and details of readmissions.

The Epworth HealthCare data were then linked to the Australian Orthopaedic Association National Joint Replacement Registry (AOANJRR) to identify those patients who had been revised for complications related to the index procedure that may have occurred outside Epworth HealthCare. The AOANJRR has collected data on almost 100% of hip arthroplasties performed in Australia and the data are externally validated against patient-level data provided by all Australian state and territory health departments. A sequential matching process is used to identify any missing data and this is subsequently retrieved by contacting the relevant hospital. Each month, in conjunction with internal validation and data quality checks, all primary procedures are linked to any subsequent revision involving the same patient, same joint, and same side. Data are also matched bi-annually with the Australian Government’s National Death Index to obtain information on the date of death. Linking revision and death to the primary procedure enables revision rates to be determined.

The primary end point was the frequency of complications defined as return to theatre as inpatient or re-admission within 30 days. The secondary end point was revision surgery. Complication rate was defined as the number of complications per total number of operations conducted using each surgical approach. Revisions were defined as reoperations of previous hip replacements where one or more of the prosthetic components were replaced, removed, or one or more components are added. Revision rate was defined as the number of revisions performed for each complication, per total number of operations conducted using each surgical approach.

## Statistical analysis

Percentages for the presence of each complication and indications for revision were statistically compared using Fisher’s exact test extended to r by c (in this case 3 by 2, three approaches by complication present/absent, or revision present/absent within complications) tables (Agresti, 2013; Mehta & Patel, 1983). All statistical analyses were conducted using Stata version 15 (Stata Corporation, College Station, Texas, 2017).

## Results

A total of 2437 THAs were performed during the study time period and were matched to records in the AOANJRR. Of these, 949 (38.9%) were performed via the anterior approach, 618 (25.4%) via the lateral approach, and 870 (35.7%) via the posterior approach. The average age of patients in this study was 66.6 years (range 19–97 years). There were 1074 male and 1363 female patients.

There were 72 (3%) patients who had complications requiring re-admission or re-operation. Peri-prosthetic fracture, dislocations and surgical site infection (SSI) were the most common complications leading to re-admission and / or re-operation (Table 1). The prevalence of complications is presented with the different volumes by approach taken into account. In our study, there was no statistically significant differences in the rates of complications between the 3 different approaches (*p* = 0.99).

**Table 1:** Complication requiring readmission

	Anterior (*n* = 949)	Lateral (*n* = 618)	Posterior (*n* = 870)	*p* value^1^	Total (*n* = 2437)
Peri-prosthetic Fracture	13 (1.4%)	9 (1.5%)	7 (0.8%)	.43	29 (1.2%)
Early stem subsidence	3 (0.3%)	1 (0.2%)	0	.31	4 (0.16%)
Dislocation	5 (0.5%)	1 (0.2%)	8 (0.9%)	.17	14 (0.6%)
SSI	2 (0.2%)	4 (0.6%)	6 (0.7%)	.24	12 (0.5%)
Haematoma	2 (0.2%)	1 (0.2%)	0	.48	3 (0.1%)
Venous thromboembolic event	3 (0.3%)	1 (0.2%)	3 (0.3%)	.90	7 (0.3%)
Neurovascular injury	0	0	1 (0.1%)	.61	1 (0.04%)
Cerebrovascular accident	1 (0.1%)	1 (0.2%)	0	.72	2 (0.08%)
Total per approach	29 (3.1%)	18 (2.9%)	25 (2.9%)	.99	72 (3.0%)

^1^Fisher’s exact test for r (approach) by c (complication present/absent) tables

Of the cases with complications detected, 28 (1.1% of all THAs) required revision due to the complications of fracture, early subsidence, dislocation and surgical site infection within the first 30 days of the index surgery. These are presented in Table 2.The overall rates of revision for the anterior, lateral and posterior approaches combined due to complications occurring within 30 days of index surgery did not significantly differ (*p* = 0.17).

**Table 2:** Revision rates within complications

	Anterior (*n* = 949)	Lateral (*n* = 618)	Posterior (*n* = 870)	*p* value^1^	Total (*n* = 2437)
Peri-prosthetic Fracture	9 (0.9%)	0	1 (0.1%)	**.003**	10 (0.4%)
Early stem subsidence	3 (0.3%)	1 (0.2%)	0	0.3	4 (0.2%)
Dislocation	3 (0.3%)	1 (0.2%)	4 (0.5%)	0.68	8 (0.3%)
SSI	1 (0.1%)	3 (0.5%)	2 (0.2%)	.35	6 (0.2%)
Total per approach	16 (1.7%)	5 (0.8%)	7 (0.8%)	0.17	28 (1.1%)

^1^Fisher’s exact test for r (approach) by c (revision present/absent within complication) tables

Logistic regression by surgical approach alone shows that overall difference in revision between approaches is not significant (*p* = 0.48). When including age and gender, the overall effect of approach is not statistically significant (*p* = 0.052), and age is not significantly related. However, the effect of gender approaches statistical significance (*p* = 0.073), with females more likely to have revisions (Odds Ratio = 1.60, 95% CI = 0.96 to 2.69) than males.

### Peri-prosthetic fractures

There were 29 cases of PPFs and 21 occurred in female patients. The overall mean age was 69.6 years, (range: 51–91 years). The body mass index (BMI) was greater than 30 kg/m^2^ in 8 (27.6%) of the 29 cases, with a mean of 27.9 kg/m^2^ and a standard deviation of 8.5 kg/m^2^ (range: 16.4–50.7 kg/m^2^).

Of the 29 cases, 19 (65.5%) underwent fixation of the fracture. These fractures were detected intra-operatively and the fixations were performed at the time of the index surgery. None of these patients went on to require subsequent revision.

There was a significantly higher rate of revision for PPF for patients undergoing the anterior approach. Nine patients (0.9% of 949 Anterior approaches) were revised for PPF, compared with none of the lateral group and 1 (0.1% of 870 patients) in the posterior group. (*p* = 0.003). Seven of those revised in the anterior group were readmitted.

Amongst the 29 patients who had PPFs, 19 patients had cement-less femur prostheses (65.5%). These 19 prostheses consisted of various models from different companies. Six patients had cemented prostheses and 3 patients had modular prostheses.

### Early subsidence

There were 4 cases of early stem subsidence without fracture, which occurred within 30 days of index surgery; 3 occurred in female patients. The mean age was 65.6, standard deviation 6.8 years (range: 58–74 years). The mean BMI was 28.6, with a standard deviation of 2.7 kg/m^2^ (range: 25.5–32 kg/m^2^). Only 1 patient had a BMI more than 30 kg/m^2^.

These patients presented with limb length discrepancy or instability. All 4 cases required revision of the femoral implant. Three of the patients were in the anterior group and 1 in the lateral group. In 2 of the patients in the anterior group, the subsequent stems utilised in the revision were 4 sizes larger than that of the stems used in the index surgeries. In the single patient in the lateral group, the stem was revised to cemented femur prosthesis.

### Dislocations

There were 14 cases of hip dislocation requiring readmission or reoperation and 11 occurred in female patients. The average age was 66.5 years, standard deviation 10.7 years (range: 47–80 years). The BMI was > 30 kg/m^2^ in 5 of the patients, with an average of 28.6 kg/m^2^, standard deviation of 7.5 kg/m^2^ (range: 17.2–43.6 kg/m^2^).

The rates of dislocation in our study for anterior, lateral and posterior approaches were 0.5, 0.2 and 0.9% respectively. These dislocation rates were not statistically significant. Eight out of 14 of the patients who had an early dislocation required revision. The revision rates for dislocation across the three approaches were not statistically significant as well. The remaining 6 patients who had dislocations which did not require revision had relocations performed in the emergency department or under anaesthesia in the operating theatre, with no subsequent sequelae.

### Surgical site infection (SSI)

There were 12 cases of SSIs. There were 7 female patients and 5 male patients, with an average age of 68.3 years and standard deviation of 14.6 years (range: 35–93 years). The BMI was greater than 30 kg/m^2^ in 4 of the patients, and the average BMI was 29.1 kg/m^2^, with a standard deviation of 5.2 kg/m^2^ (range: 20.3–39.5 kg/m^2^).

Nine patients had deep SSIs requiring reoperation, whilst the remaining 3 had superficial SSIs which resolved with conservative management with antibiotics.

Six patients with SSI required removal and revision of the implant. The revision rate for SSIs across the 3 approaches was not statistically significant. Three patients had washouts without revision of implant.

### Other complications requiring reoperations

One patient who had index surgery via the anterior approach was readmitted for post-operative haematoma causing sciatic nerve compression, presenting with foot drop. The patient underwent surgery for an evacuation of the haematoma and neurolysis of the sciatic nerve via the posterolateral approach.

There was 1 case of iatrogenic injury to the sciatic nerve in a patient operated on through the posterior approach. The patient was noted to have post-operative foot drop and went on to have an exploration and repair of the sciatic nerve, which was noted to have a 30% division.

## Discussion

In regard to the primary endpoint, this study has demonstrated that, whilst the prevalence of complications following THA was not statistically different amongst the three approaches, there was a higher overall rate of revision for the anterior approach, and this was due to early revisions for PPF.

The benefits and drawbacks of the different surgical approaches for THA continue to be a source of some debate and controversy. Studies supporting the use of the anterior approach have reported shorter lengths of inpatient stay, improved clinical and functional outcomes, improved gait dynamics and a lower dislocation rate [2, 3, 12, 13]. However, other studies report a higher risk of complications especially early after surgery [14].

### Peri-prosthetic fractures

The commonest complications following THA in our study were fractures. There were a significantly higher proportion of fractures that went on to receive revision in the anterior approach group. If the fractures were detected and fixed intra-operatively, there were no revisions but if they occurred intra-operatively and were not recognised, then there was a higher revision rate. This was the case despite the use of fluoroscopy for the anterior approach in almost all the cases.

The fractures in the anterior group occurred in the calcar, and involved cement-less tapered wedge femoral prostheses, which were most commonly used in the anterior approach. The study by De Geest’s group et al. [6] showed the fractures in their study involved a single stem design. The effect of stem designs in different approaches was investigated by Panichkul et al. [15] and a higher stem revision rate was found in the anterior approach group utilising a tapered wedge stem; aseptic loosening and peri-prosthetic fractures occurred in this group compared to none in the posterior and lateral approach groups.

It is not known if the increased risk of fractures in the anterior approach can be attributed more to the approach itself, or the limitations of the implants or instrumentation. It has been theorised that the supine positioning, with the usage of traction tables and manipulation of the lower limb for surgical exposure, may result in higher forces during elevation of the femur and broaching [16]. These can possibly increase hoop stresses at the calcar and when combined with certain prostheses designs, result in calcar fractures. The use of a positioning table itself has been studied [16] and not found to be an independent risk factor for femoral fractures.

It is probable that factors such as the positioning of the patient and femur, amount of exposure and instrumentation, while individually not causal factors of statistical significance, combine to elevate the risk of iatrogenic fractures above that of the posterior and lateral approach. Meneghini et al. [17]found that the greatest proportion of early femoral peri-prosthetic fractures or loosening occurred in the anterior approach. A multivariate analysis controlling for gender, age, BMI, laterality, bilateral versus unilateral procedure, bone quality and femoral stem type did not reveal any significant influence on femoral component loosening, which may indicate that a complex interplay of factors leads to a combined effect on the increased likelihood of such complications in the anterior approach.

Seven of the fractures in the anterior group (53.8% of the fractures in the anterior group) were not detected on intra-operative imaging. This did not occur in the lateral or posterior approaches. This is again similar to the findings of De Geest et al., where peri-prosthetic fractures were only detected post-operatively on checking radiographs or being readmitted from rehabilitation. Malek et al. [18] also encountered the same in their study which had a 2.6% rate of occult intra-operative peri-prosthetic femoral fracture in the anterior approach, which was not identified on intra-operative image intensifier screening. This may point to technical limitations in the imaging quality of intra-operative imaging. Intra-operative imaging is utilised to check cup positioning and limb length, and a subtle cortical crack may go unnoticed by the surgeon who focused on the former. Surgeons who encountered complications were interviewed and felt that an intra-operative crack may have occurred on stem insertion and therefore was not detected with imaging.

The two patients with early subsidence without fractures required revision with stems which were significantly larger than those initially inserted. In absence of fractures, this appears to indicate that the limitations of current instrumentation and surgical exposure may cause issues during broaching in this approach. It is postulated that the surgeons had difficulty inserting the broach directly down the intramedullary canal, causing an inadequate filling of the canal and thus incorrect sizing of the femoral components.

### Dislocations

The posterior approach is known to have a slightly higher risk of dislocations, whilst the anterior and lateral approaches allow for preservation of the posterior soft tissue envelope. Dislocation rates reported in literature for the posterior approach range from 1 to 5% [19], whilst that of the anterior approach ranges from 0.61 to 1.5% [20, 21] and that of the lateral approach ranges from 0.4 to 0.55% [11, 22]. Of interest is Meneghini’s [17] finding in that, although the majority of revisions for instability occurred in the posterior approach (47.5%), the revision rate for instability in the anterior group was 37.5%. Other studies involving matched cohorts and a registry report of 11,112 matched registry cases revealed no difference in dislocation rates between the anterior approach and posterior approach [23, 24], with the authors concluding that the posterior approach, with capsular repair, and the use of larger femoral heads, has decreased the risk of dislocation to a level comparable to that of the anterior approach.

The rate of dislocation in our study parallels that in literature, with the rates for anterior, lateral and posterior approaches being 0.5, 0.2 and 0.9% respectively. Revision rates due to dislocations did not differ significantly across the 3 approaches. Again, whilst the numbers are small, the heterogeneity of the study population does indicate that in the real world setting, the risk of dislocation is not completely removed by the anterior approach, and attention on the surgeon’s part with respect to acetabular cup and femoral stem positioning is still paramount, regardless of approach. As with fracture complications, neither gender nor BMI appeared to be risk factors for instability.

### SSIs and haematomas

The anterior approach has been linked to an increased re-operation rate due to wound complications, such as haematomas with or without infection, delayed wound healing or prosthetic joint infections [25]. This has been attributed to the thinner skin in the proximal thigh as compared to the lateral thigh, the proximity to the waist crease and the increased skin tension across the flexion crease causing raised shear stresses [4].

A study by Jahng [26] has looked into risk factors for wound complications after THA by an anterior approach. Their findings were that a BMI of > 30 kg/m^2^ and diabetes placed patients at greater risk for wound complications and re-operation. This may serve as a guide to surgeons when considering the suitability of patients for the anterior approach.

In our patients, only 4 out of 12 patients had BMI > 30 kg/m^2^ but the low numbers did not allow us to analyse if BMI was a risk factor for wound complications. Among the patients in which re-operation was required for SSI, there did not appear to be any approach associated with an increased risk of SSI. In addition, whilst half of the patients with SSIs went on to require revision of implants, there was no significant difference in revision rate for infection among approaches.

### Venous thromboembolic events

There has been no recent literature comparing the rates of venous thromboembolic events (VTE) among the approaches and in our patients, VTE occur in the different approaches. Five out of 7 of the patients who had VTE were female. In our patients, whist BMI was more than 30 kg/m^2^ for 4 patients out of 7, with an average of 31.6 kg/m^2^. However, the numbers are too low to allow analysis if gender or BMI are significant risk factors. Thus the incidence of VTE in patients undergoing THA in our institution appears to be a function of known risk factors as established in literature as opposed to being approach specific.

There are several strengths to this study. It examines the overall complications of all THA performed at a large tertiary hospital group and includes the results of all the surgeons performing arthroplasty, and not just from a specialized joint arthroplasty centre. All procedures were linked to the AOA NJRR to capture all revisions including those that may not have occurred at the hospital. A case record review was performed of all complications as distinct from relying on information from an administrative dataset review. There are also limitations to this study. Although data are collected on length of time of surgery the setup for the anterior hip approach with a traction table and fluoroscopy is recorded within the whole theatre time. Therefore this may have lengthened the time of the operation recorded for the anterior approach compared to that for patients receiving the lateral or posterior approaches. Because of this we were unable to directly examine whether length of surgery is correlated with complications. We also did not analyse the data by surgeon experience and are aware of the effect of the learning curve on the rate of revision for the anterior approach to the hip [8]. However, the study period was well after the introduction of the anterior approach and the majority of surgeons in the group were experienced in anterior approach to the hip. Finally, data regarding femoral head sizes were not consistently recorded for all patients and analysed in this study with regards to dislocation rates across the 3 surgical approaches.

## Conclusion

The complication rates do not differ across the 3 surgical approaches for THA. However, at our institution, revision rate for PPF, in the first 30 days after the index surgery are significantly higher in the anterior approach compared with the posterior and lateral approaches. Surgeons utilising this approach should be vigilant for femoral fractures, and pay careful attention to the surgical exposure and to intra-operative imaging.

## Data Availability

The datasets used and/or analysed during the current study are available from the corresponding author on reasonable request.

## Abbreviations

AOANJRR: Australian Orthopaedic Association National Joint Replacement Registry
BMI: Body mass index
PPF: Peri-prosthetic fracture
SSI: Surgical site infection
THA: Total hip arthroplasty
